# *Candida* Vaginitis: When Opportunism Knocks, the Host Responds

**DOI:** 10.1371/journal.ppat.1003965

**Published:** 2014-04-03

**Authors:** Brian M. Peters, Junko Yano, Mairi C. Noverr, Paul L. Fidel

**Affiliations:** 1 Department of Oral and Craniofacial Biology, Dental School, Louisiana State University Health Sciences Center, New Orleans, Louisiana, United States of America; 2 Department of Prosthodontics, Dental School, Louisiana State University Health Sciences Center, New Orleans, Louisiana, United States of America; 3 Department of Microbiology, Immunology, and Parasitology, School of Medicine, Louisiana State University Health Sciences Center, New Orleans, Louisiana, United States of America; University of Notre Dame, United States of America

## Introduction

*Candida albicans*, an opportunistic polymorphic fungus and resident of the normal vaginal microbiota, is the leading causative agent of vulvovaginal candidiasis (VVC) and presents major quality of life issues for women worldwide [1]. *Candida* vaginitis is characterized by itching, burning, pain, and redness of the vulva and vaginal mucosa and often accompanied by vaginal discharge. Predisposing factors for primary VVC include high-estrogen oral contraceptive use, hormone replacement therapy, antibiotic usage, and underlying diabetes mellitus. It is estimated that 75% of all women of childbearing age will be afflicted by VVC at least once in their lifetime [2]. Of these, approximately 5–8% (approximately 150 million worldwide) suffer from recurrent VVC (RVVC), resulting in idiopathic chronic episodes of vaginal irritation that require antifungal maintenance therapy (e.g., azoles) to partially control symptoms [1]. Although these treatments are typically effective at reducing organism burden and symptoms, the static function of azole activity and fungal recalcitrance to clearance are key factors resulting in recurrence [3]. It is proposed that RVVC and VVC both involve similar immunopathologies but that the triggers occur with greater sensitivity in individuals with RVVC. Continuously rising vaginitis-related healthcare costs are estimated at $1.8 billion annually in the United States alone [4]. These unsustainable costs further necessitate a comprehensive understanding of vaginitis and the host and fungal factors that contribute to its immunopathology.

### The Role of Host Immunity in *Candida* Vaginitis: Historical and Contemporary Perspectives

Susceptibility to oral, chronic mucocutaneous, and gastrointestinal candidiasis has been clearly linked to deficiencies in cell-mediated immunity (CMI) [5]. Therefore, susceptibility to *Candida* vaginitis was also long believed to result from defects in the adaptive immune response. However, numerous clinical studies examining women with RVVC and the use of an experimental mouse model to evaluate roles for CMI or humoral immunity (HI) revealed no appreciable protection provided by local or systemic adaptive immune mechanisms [6], [7], [8]. In support of these findings, relatively high production of immunoregulatory factors (e.g., TGF-β, T-regs, and Υ/δ T-cells) in the vagina may partly explain the lack of functional local CMI [9], [10]. Despite a lack of supportive evidence for a role of adaptive immunity in vaginitis, the newly characterized Th17 axis of CMI, which links innate and adaptive immune responses, has been shown to be critical for local protection against oropharyngeal candidiasis (OPC) [11]. Accordingly, animal models were used to determine its potential role in mucosal immunity during vaginitis. However, discrepant findings amongst mouse models have led to contradictory conclusions: one study using a less stringent pharmacologic approach to Th17 blockade demonstrated a modest role for Th17 involvement [12], while a more rigorous approach using Th17 axis-knockout mice showed no such function [12], [13]. Thus, the role of Th17 responses during vaginitis remains unresolved and lacks any supportive clinical evidence. As for mucosal HI, some animal models have demonstrated modest antibody-mediated protection against vaginitis [14]. It is conceivable that protective human antibodies do exist but occur naturally at concentrations in vaginal secretions too low to sufficiently mediate protection.

**Figure 1:**
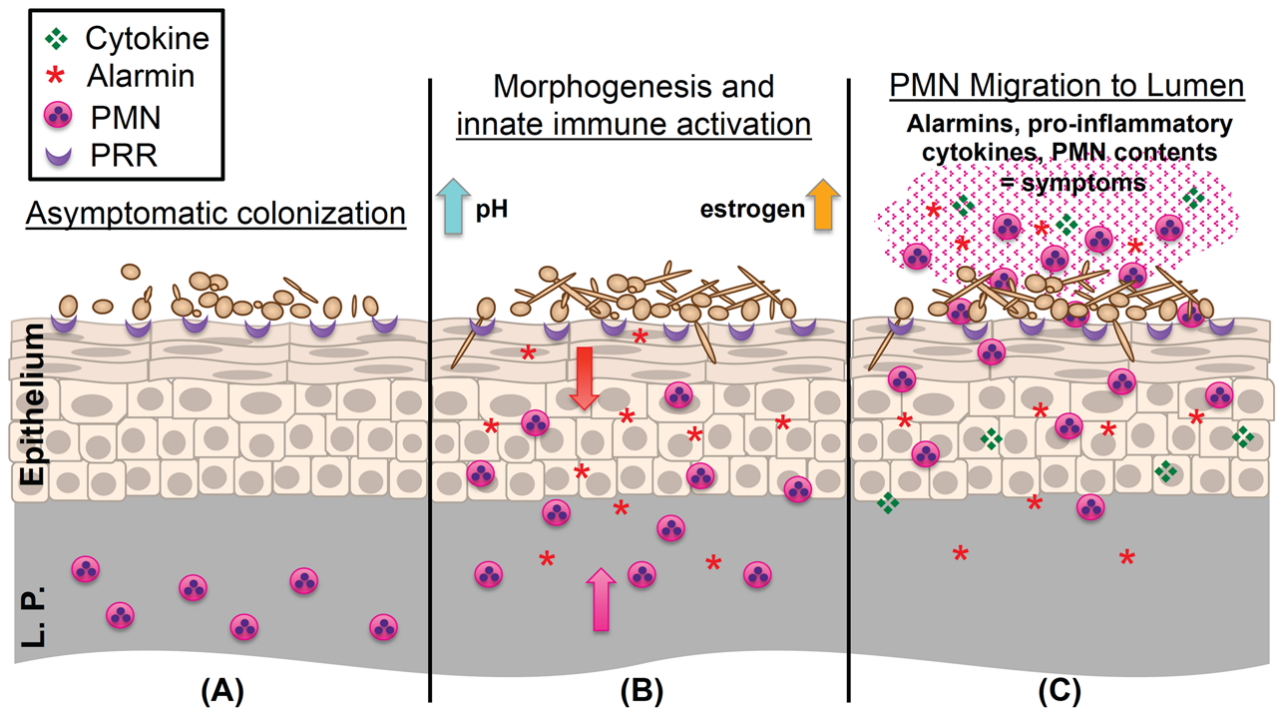
Working model of the immunopathogenesis of *C. albicans* vaginitis. (**A**) Yeast forms of *C. albicans* asymptomatically colonize the vaginal epithelium despite the presence of numerous pattern recognition receptors (PRR) on the epithelial surface. (**B**) *C. albicans* begins to undergo the yeast-to-hypha switch under morphogenesis-inducing conditions (increases in estrogen, elevated vaginal pH, and microbiota disruption). Augmented recognition by PRRs, increased hyphal biomass, and expression of hypha-associated virulence factors elicits inflammatory signaling (S100A8/9 alarmins and proinflammatory cytokines) in the vaginal epithelium, resulting in initial migration of PMNs from the lamina propria (L.P.) to the vaginal lumen. (**C**) Failure to adequately reduce immunopathological triggers results in the continued expression of innate immune effectors by the vaginal epithelium. These initial signals, coupled with secondary amplification of immune effectors by recruited PMNs, contribute to symptomatic infection and characteristic immunopathology.

While exhaustive efforts have found no major role for adaptive immunity in susceptibility to vaginitis, recent studies have identified the importance of innate immunity in regulating vaginitis symptomatology. A paramount study using women volunteers challenged with live *C. albicans* determined that vaginitis symptoms were associated with polymorphonuclear leukocyte (PMN) recruitment into the vagina and that organism burden alone was not predictive of disease [15]. Moreover, depletion of PMNs in mice did not result in increased fungal load but did decrease histological evidence of vaginal inflammation [16], [17]. Most recently, a family of calcium-binding proteins termed S100A8 and S100A9 “alarmins” have been implicated in the innate vaginal epithelial cell response to *C. albicans* (see Figure 1) [18]. Because these proteins have vigorous PMN chemotactic activity, it was hypothesized that epithelial expression of S100s may play a key role in controlling PMN migration into the vaginal lumen. However, while this was confirmed, studies using mice lacking expression of S100A8/9 proteins determined that these factors were sufficient but not necessary for driving the PMN response [19]. Collectively, these exciting new studies highlight the immunopathological response as a crucial element of vaginitis pathogenesis. Future clinical studies, however, are required to confirm the presence and function of alarmins during human infection.

As alluded to above, resultant findings from animal models must be translatable to the human host. One important point to consider is that laboratory rodents, unlike humans, do not naturally harbor *C. albicans* as commensal organisms. Although the estrogen-dependent mouse model of vaginitis closely mimics clinical infection, observed antifungal immune responses are considered primary and may be exaggerated as compared to human infection, in which repeated exposure, immunotolerance, or higher signaling thresholds to *Candida* may be encountered. Despite this shortcoming, the mouse model of vaginitis has been an indispensible tool for dissecting the immunological mechanisms associated with this highly complex disease.

### Genetic Susceptibility to Fungal Infections: Applied Lessons to Vaginitis?

Several clinical studies have been designed to identify genetic polymorphisms in factors with putative importance for antifungal defense, such as the pathogen recognition receptors (PRRs) Dectin-1, Dectin-2, MINCLE (macrophage-inducible C-type lectin), TLR2/4 (Toll-like receptors 2 and 4), mannose binding lectin (MBL), mannose receptor (MR), and DC-SIGN/SIGNR1 (Dendritic Cell-Specific Intercellular adhesion molecule-3-Grabbing Non-integrin) [20]. Ligation of these receptors to their cognate ligands (typically *C. albicans* cell-wall components) induces proinflammatory signaling, initiating recruitment of both innate and adaptive immune cells to elicit potentially protective responses. Data acquired from associative genetic studies and their relevance to vaginitis pathogenesis is discussed below.

A homozygous polymorphism (Tyr238X stop codon) in the C-type lectin receptor (CLR) Dectin-1 (β-glucan recognition) confers susceptibility to chronic onychomycosis and RVVC but not OPC [21]. Studies conducted by Yano et al. surprisingly demonstrated lack of expression of Dectin-1 on murine vaginal epithelium and wild-type levels of neutrophil recruitment and fungal burden in Dectin-1-/- mice during vaginal infection [19]. However, simultaneous blockade of TLR4 and SIGNR1 signaling reduced S100 alarmin expression in vaginal epithelial cells when challenged with *C. albicans*, suggesting an important function of these cell-surface receptors at the vaginal mucosa. Whereas the role of Dectin-1 during *Candida* vaginitis remains inconclusive, these other PRRs may serve more prominent roles. Signal transduction via CLRs converges on the common signaling adaptor CARD9. A homozygous point mutation (Q295X) abolishing CARD9 function has been associated with increased risk of fungal infection, including OPC and RVVC [22]. Unfortunately, lack of evidential expression data for Dectin-1 and CARD9 in the vaginal epithelium provokes further questions on their relevance in vaginitis pathogenesis. Functional defects in MBL and MR have also been suggested to mediate susceptibility to fungal infections. These host factors act by neutralizing or recognizing the carbohydrate mannan found ubiquitously on the surface of fungi. A series of clinical studies have elucidated an association between a polymorphism in the MBL-2 gene (mutant codon 54) and susceptibility to RVVC [23], [24]. While these findings are of significant clinical interest, protective roles for MBL and MR against *Candida* in the vagina remain unclear due to lack of supportive mechanistic studies.

Despite important new information gained from these studies, more in-depth molecular and immunological research is urgently required. While cell-surface receptors and signaling adaptors undoubtedly play a role during vaginal fungal infection, discrepancies between animal models and human clinical data further cloud the overall picture of vaginitis pathogenesis. Careful consideration must be taken to avoid overinterpretation of limited clinical datasets or mischaracterization of animal model data not relevant to the clinical scenario. Furthermore, the highly compartmentalized nature of immune responses to *C. albicans* at different anatomical sites warrants discretion in proposing overarching hypotheses without detailed experimentation.

### *Candida albicans* Is a Robust Opportunistic Fungal Pathogen

Several properties of *C. albicans* have been proposed to play major roles in causing disease: morphogenesis, secreted factors, and biofilm formation. As a polymorphic fungus, *C. albicans* adopts two major morphological forms, the commensalistic ovoid yeast and the pathogenic filamentous hypha. The transition from yeast to hypha is under the control of a complex set of environmental sensors and has long been considered to be the major virulence factor of this pathogen [25]. In vitro studies utilizing *Candida*-infected, reconstituted human vaginal epithelium demonstrated a requirement for hyphae to uniquely induce innate immune signaling [26]. These findings were supported by challenging mice intravaginally with isogenic *C. albicans* strains, some of which were defective in key transcriptional regulators mediating the yeast-to-hypha switch. Despite similar colonization among strains, those defective in hypha formation displayed significantly reduced immunological markers of vaginitis symptomatology (including reduced PMNs and S100A8) [17]. Cumulatively, these studies confirm an important role for the morphogenetic response in vaginitis immunopathology. These points are illustrated in Figure 1 by yeast cells that asymptomatically colonize the vaginal epithelium despite being recognized by cell-surface PRRs. The yeast-to-hypha transition further engages additional PRRs, causes mechanical disruption of the epithelial surface, and augments expression of fungal virulence factors. Ultimately, these cellular insults result in damage-mediated responses leading to the recruitment of PMNs, culminating in vaginitis immunopathology.

Aside from the yeast-to-hypha switch, *C. albicans* also produces a number of secretory products that have been implicated in the pathogenesis of vaginitis. A family of homologous secreted aspartyl proteinases (SAPs) has demonstrated differential expression during vaginitis [27]. SAPs act by cleaving proteins on host epithelium, resulting in mucosal structural damage and enhanced fungal burden. Although not characterized in as much detail, candidal lipases have also been implicated in disease pathology [28].

Another major virulence property of *C. albicans* is its ability to form biofilms on both biotic and abiotic surfaces. Biofilms are complex, highly organized, three-dimensional structures formed by communities of microbes conferring properties of increased adhesion, recalcitrance to clearance by the host immune system, and enhanced antimicrobial resistance. It has been demonstrated that *C. albicans* forms biofilms on the vaginal mucosa of mice but biofilm formation is not required for colonization [29]. Furthermore, because morphogenesis and biofilm formation are intimately linked, it is unclear what role biofilms play in the context of immunity.

### Vaginitis: A Multifactorial Disease

Other biologically relevant criteria are also critical in initiating disease pathology. The effects of estrogen on the vaginal mucosa appear to be crucial for vaginitis progression. Production of estrogen during the menstrual cycle causes the keratinized vaginal epithelium to cornify, thicken, and eventually slough. In fact, it is during these periods of high estrogenic activity that women are most susceptible to vaginitis [30]. This is further evidenced by the fact that prepubescent girls and postmenopausal women (low-estrogen producers) rarely develop vaginitis. However, postmenopausal women on hormone replacement therapy frequently become resusceptible [30]. Indeed, animal models of vaginitis almost always include administration of exogenous estrogen to maintain fungal burdens, emphasizing the importance of estrogen in disease pathology (Figure 1) [31]. Aside from physiological changes, estrogen also exhibits immunomodulatory effects, including decreased antimicrobial peptide expression, modulated PRR expression, reduced antigen presentation, decreased T cell priming, diminished mucosal antibody production, enhanced antigenic tolerance, and impairment of Th17 signaling [32], [33], [34]. Not only does estrogen have pleiotropic effects on the host, it also directly affects *C. albicans* itself. Estrogen enhances *Candida* adherence to vaginal epithelial cells, augments germ tube formation, and increases hyphal length [35], [36]. Furthermore, *C. albicans* encodes an estrogen binding protein (EBP), recently revealed to act as an oxidoreductase [37]. Inhibition of EBP by estrogen results in increased expression of the drug efflux pumps CDR1/2, antimicrobial resistance mechanisms employed by fungi against commonly used azoles [35]. Estrogen has also been shown to up-regulate expression of the fungal heat-shock protein Hsp90, conferring resistance to biological and chemical stressors [38]. Together, these estrogen-induced modifications of host and fungal responses may partly explain lack of protective immunity to vaginitis and recalcitrance to clearance.

The pH of the vaginal microenvironment and composition of the bacterial microbiota are also important for disease pathogenesis. The average adult human vaginal pH (4.5) is maintained at an acidic level to aid in the inhibition of microorganism overgrowth. A low vaginal pH inhibits the yeast-to-hypha switch in *C. albicans* [39]. Therefore, increases in pH levels are believed to promote vaginitis (Figure 1). Interestingly, mice maintain a neutral vaginal pH that favors hyphal formation, thus making them a robust model system for *Candida* vaginitis. The bacterial microbiota are also important in maintaining a healthy vaginal environment, as antibiotic administration is a major risk factor for developing vaginitis, presumably through disruption of the natural bacterial communities existing at the mucosal interface [40]. Decreased levels of ubiquitous acid-producing lactobacilli lead to increased vaginal pH levels and augmented colonization of potential pathogens, including *C. albicans*.

### New Frontiers in *Candida* Vaginitis and Closing Remarks

A comprehensive understanding of the immune response to *Candida* vaginitis is far from complete. The fields of mucosal biology and immunology are rapidly expanding and may provide insights into this complex disease. Recent evidence suggests that danger–signal-mediated inflammasome signaling (i.e. processing, cleavage, and secretion of IL-1β) plays an important role in the innate response to various pathogens at mucosal surfaces, including *C. albicans* [17], [41], [42], [43]. However, more research is needed to address the role (if any) of inflammasome signaling during vaginitis and whether inflammasome activation may be linked to S100A8/9 production or PMN infiltration. In any case, novel immunomodulatory therapies may be viable options for curtailing vaginitis immunopathology, especially in RVVC.

Development of accurate diagnostics and preventive vaccines remains a priority. However, these developments do not come without significant challenges. An inexpensive, simple, and rapid diagnostic test that can simultaneously detect symptomatology (e.g., innate immune effectors) along with fungal presence is the ultimate goal in order to distinguish asymptomatic colonization from true infection. It is also imperative that such a diagnostic will discriminate between vaginitis of varying etiology (including those of fungal, bacterial, and protozoal origin) so that appropriate antimicrobial therapy may be administered without delay. Additionally, preliminary clinical trials of two antifungal vaccines (NDV-3, based on the recombinant candidal adhesin Als3, and PEV7, which utilizes SAP-2 as the immunogen) have provided promising results, efficaciously reducing fungal burden during intravaginal challenge with *C. albicans* [44], [45]. However, advanced clinical trials will be required to determine if these vaccines can reduce fungal burdens adequately to eliminate the triggers for vaginitis immunopathology in both VVC and RVVC populations.

Vaginitis is a complex disease, requiring a “perfect storm” scenario to initiate infection. Estrogen production, microbiota disruption, pH modification, fungal virulence factor expression, and exuberant innate immune activity must synchronize to culminate in symptomatic infection. Current research has elucidated a major paradigm shift in the philosophy of *Candida* vaginitis pathogenesis, highlighting the role of the host innate immune response in disease immunopathology. Moreover, another paradigm change is emerging that is shifting the focus on fungal burden as the sole outcome phenotype in the context of understanding virulence mechanisms and developing novel treatment strategies toward focusing on the immunopathological response instead. Accordingly, although much insight has been gained from extensive studies in this field, scientific endeavors aimed at preventive therapies, improved diagnostics, and pathogenesis will require careful attention to the immunopathogenic response to ultimately combat this highly significant, opportunistic fungal disease.

## References

[1] Achkar JM, Fries BC (2010) *Candida* infections of the genitourinary tract. Clin Microbiol Rev 23: 253–273. 20375352 10.1128/CMR.00076-09 PMC2863365

[2] Sobel JD (1997) Vaginitis. N Engl J Med 337: 1896–1903. 9407158 10.1056/NEJM199712253372607

[3] Sobel JD, Zervos M, Reed BD, Hooton T, Soper D, et al (2003) Fluconazole susceptibility of vaginal isolates obtained from women with complicated *Candida* vaginitis: clinical implications. Antimicrob Agents Chemother 47: 34–38. 12499165 10.1128/AAC.47.1.34-38.2003 PMC148960

[4] Foxman B, Barlow R, D'Arcy H, Gillespie B, Sobel JD (2000) *Candida* vaginitis: self-reported incidence and associated costs. Sex Transm Dis 27: 230–235. 10782746 10.1097/00007435-200004000-00009

[5] Ashman RB (2008) Protective and pathologic immune responses against *Candida albicans* infection. Front Biosci 13: 3334–3351. 18508436 10.2741/2929

[6] Fidel PL Jr, Lynch ME, Redondo-Lopez V, Sobel JD, Robinson R (1993) Systemic cell-mediated immune reactivity in women with recurrent vulvovaginal candidiasis. J Infect Dis 168: 1458–1465. 8245529 10.1093/infdis/168.6.1458

[7] Fong IW, McCleary P, Read S (1992) Cellular immunity of patients with recurrent or refractory vulvovaginal moniliasis. Am J Obstet Gynecol 166: 887–890. 1550158 10.1016/0002-9378(92)91356-f

[8] Mendling W, Koldovsky U (1996) Investigations by cell-mediated immunologic tests and therapeutic trials with thymopentin in vaginal mycoses. Infect Dis Obstet Gynecol 4: 225–231. 18476097 10.1155/S1064744996000439 PMC2364501

[9] Taylor BN, Saavedra M, Fidel PL Jr (2000) Local Th1/Th2 cytokine production during experimental vaginal candidiasis: potential importance of transforming growth factor-beta. Med Mycol 38: 419–431. 11204879 10.1080/mmy.38.6.419.431

[10] Wormley FL Jr, Steele C, Wozniak K, Fujihashi K, McGhee JR, et al (2001) Resistance of T-cell receptor delta-chain-deficient mice to experimental *Candida albicans* vaginitis. Infect Immun 69: 7162–7164. 11598094 10.1128/IAI.69.11.7162-7164.2001 PMC100112

[11] Conti HR, Shen F, Nayyar N, Stocum E, Sun JN, et al (2009) Th17 cells and IL-17 receptor signaling are essential for mucosal host defense against oral candidiasis. J Exp Med 206: 299–311. 19204111 10.1084/jem.20081463 PMC2646568

[12] Pietrella D, Rachini A, Pines M, Pandey N, Mosci P, et al (2011) Th17 cells and IL-17 in protective immunity to vaginal candidiasis. PLoS ONE 6: e22770. 21818387 10.1371/journal.pone.0022770 PMC3144947

[13] Yano J, Kolls JK, Happel KI, Wormley F, Wozniak KL, et al (2012) The acute neutrophil response mediated by S100 alarmins during vaginal *Candida* infections is independent of the Th17-pathway. PLoS ONE 7: e46311. 23050010 10.1371/journal.pone.0046311 PMC3457984

[14] de Bernardis F, Santoni G, Boccanera M, Spreghini E, Adriani D, et al (2000) Local anticandidal immune responses in a rat model of vaginal infection by and protection against *Candida albicans* . Infect Immun 68: 3297–3304. 10816477 10.1128/iai.68.6.3297-3304.2000 PMC97585

[15] Fidel PL Jr, Barousse M, Espinosa T, Ficarra M, Sturtevant J, et al (2004) An intravaginal live *Candida* challenge in humans leads to new hypotheses for the immunopathogenesis of vulvovaginal candidiasis. Infect Immun 72: 2939–2946. 15102806 10.1128/IAI.72.5.2939-2946.2004 PMC387876

[16] Black CA, Eyers FM, Russell A, Dunkley ML, Clancy RL, et al (1998) Acute neutropenia decreases inflammation associated with murine vaginal candidiasis but has no effect on the course of infection. Infect Immun 66: 1273–1275. 9488427 10.1128/iai.66.3.1273-1275.1998 PMC108047

[17] Peters BM, Palmer GE, Fidel PL Jr, Noverr MC (2014) Fungal morphogenetic pathways are required for the hallmark inflammatory response during *Candida* vaginitis. Infect Immun 82: 532–543. 24478069 10.1128/IAI.01417-13 PMC3911367

[18] Yano J, Lilly E, Barousse M, Fidel PL Jr (2010) Epithelial cell-derived S100 calcium-binding proteins as key mediators in the hallmark acute neutrophil response during *Candida* vaginitis. Infect Immun 78: 5126–5137. 20823201 10.1128/IAI.00388-10 PMC2981313

[19] Yano J, Palmer GE, Eberle KE, Peters BM, Vogl T, et al (2014) Vaginal epithelial cell-derived S100 alarmins induced by *C. albicans* via pattern recognition receptor interactions is sufficient but not necessary for the acute neutrophil response during experimental vaginal candidiasis. Infect Immun 82: 783–792. 24478092 10.1128/IAI.00861-13 PMC3911366

[20] Netea MG, Brown GD, Kullberg BJ, Gow NA (2008) An integrated model of the recognition of *Candida albicans* by the innate immune system. Nat Rev Microbiol 6: 67–78. 18079743 10.1038/nrmicro1815

[21] Ferwerda B, Ferwerda G, Plantinga TS, Willment JA, van Spriel AB, et al (2009) Human dectin-1 deficiency and mucocutaneous fungal infections. N Engl J Med 361: 1760–1767. 19864674 10.1056/NEJMoa0901053 PMC2773015

[22] Rosentul DC, Plantinga TS, Oosting M, Scott WK, Velez Edwards DR, et al (2011) Genetic variation in the Dectin-1/CARD9 recognition pathway and susceptibility to candidemia. J Infect Dis 204: 1138–1145. 21881131 10.1093/infdis/jir458 PMC3164426

[23] Babula O, Lazdane G, Kroica J, Ledger WJ, Witkin SS (2003) Relation between recurrent vulvovaginal candidiasis, vaginal concentrations of mannose-binding lectin, and a mannose-binding lectin gene polymorphism in Latvian women. Clin Infect Dis 37: 733–737. 12942410 10.1086/377234

[24] Donders GG, Babula O, Bellen G, Linhares IM, Witkin SS (2008) Mannose-binding lectin gene polymorphism and resistance to therapy in women with recurrent vulvovaginal candidiasis. BJOG 115: 1225–1231. 18715406 10.1111/j.1471-0528.2008.01830.x

[25] Biswas S, Van Dijck P, Datta A (2007) Environmental sensing and signal transduction pathways regulating morphopathogenic determinants of *Candida albicans* . Microbiol Mol Biol Rev 71: 348–376. 17554048 10.1128/MMBR.00009-06 PMC1899878

[26] Moyes DL, Murciano C, Runglall M, Islam A, Thavaraj S, et al (2011) *Candida albicans* yeast and hyphae are discriminated by MAPK signaling in vaginal epithelial cells. PLoS ONE 6: e26580. 22087232 10.1371/journal.pone.0026580 PMC3210759

[27] Taylor BN, Staib P, Binder A, Biesemeier A, Sehnal M, et al (2005) Profile of *Candida albicans*-secreted aspartic proteinase elicited during vaginal infection. Infect Immun 73: 1828–1835. 15731084 10.1128/IAI.73.3.1828-1835.2005 PMC1064921

[28] Schaller M, Borelli C, Korting HC, Hube B (2005) Hydrolytic enzymes as virulence factors of *Candida albicans* . Mycoses 48: 365–377. 16262871 10.1111/j.1439-0507.2005.01165.x

[29] Harriott MM, Lilly EA, Rodriguez TE, Fidel PL Jr, Noverr MC (2010) *Candida albicans* forms biofilms on the vaginal mucosa. Microbiology 156: 3635–36344. 20705667 10.1099/mic.0.039354-0 PMC3068702

[30] Hong E, Dixit S, Fidel PL, Bradford J, Fischer G (2013) Vulvovaginal candidiasis as a chronic disease: diagnostic criteria and definition. J Low Genit Tract Dis. 10.1097/LGT.0b013e318287aced 23760143

[31] Hamad M, Abu-Elteen KH, Ghaleb M (2004) Estrogen-dependent induction of persistent vaginal candidosis in naive mice. Mycoses 47: 304–309. 15310335 10.1111/j.1439-0507.2004.00994.x

[32] Hickey DK, Fahey JV, Wira CR (2013) Mouse estrous cycle regulation of vaginal versus uterine cytokines, chemokines, alpha-/beta-defensins and TLRs. Innate Immun 19: 121–131. 22855555 10.1177/1753425912454026 PMC4322431

[33] Relloso M, Aragoneses-Fenoll L, Lasarte S, Bourgeois C, Romera G, et al (2012) Estradiol impairs the Th17 immune response against *Candida albicans* . J Leukoc Biol 91: 159–165. 21965175 10.1189/jlb.1110645

[34] Wira CR, Fahey JV, Sentman CL, Pioli PA, Shen L (2005) Innate and adaptive immunity in female genital tract: cellular responses and interactions. Immunol Rev 206: 306–335. 16048557 10.1111/j.0105-2896.2005.00287.x

[35] Cheng G, Yeater KM, Hoyer LL (2006) Cellular and molecular biology of *Candida albicans* estrogen response. Eukaryot Cell 5: 180–191. 16400181 10.1128/EC.5.1.180-191.2006 PMC1360257

[36] Hollmer C, Essmann M, Ault K, Larsen B (2006) Adherence and blocking of *Candida albicans* to cultured vaginal epithelial cells: treatments to decrease adherence. Infect Dis Obstet Gynecol 2006: 98218. 17485817 10.1155/IDOG/2006/98218 PMC1581476

[37] Madani ND, Malloy PJ, Rodriguez-Pombo P, Krishnan AV, Feldman D (1994) *Candida albicans* estrogen-binding protein gene encodes an oxidoreductase that is inhibited by estradiol. Proc Natl Acad Sci U S A 91: 922–926. 8302868 10.1073/pnas.91.3.922 PMC521425

[38] Zhang X, Essmann M, Burt ET, Larsen B (2000) Estrogen effects on *Candida albicans*: a potential virulence-regulating mechanism. J Infect Dis 181: 1441–1446. 10762574 10.1086/315406

[39] Vylkova S, Carman AJ, Danhof HA, Collette JR, Zhou H, et al (2011) The fungal pathogen *Candida albicans* autoinduces hyphal morphogenesis by raising extracellular pH. MBio 2: e00055–00011. 21586647 10.1128/mBio.00055-11 PMC3101780

[40] Ma B, Forney LJ, Ravel J (2012) Vaginal microbiome: rethinking health and disease. Annu Rev Microbiol 66: 371–389. 22746335 10.1146/annurev-micro-092611-150157 PMC3780402

[41] Basso B, Gimenez F, Lopez C (2005) IL-1beta, IL-6 and IL-8 levels in gyneco-obstetric infections. Infect Dis Obstet Gynecol 13: 207–211. 16338780 10.1080/10647440500240664 PMC1784579

[42] Matzinger P (2002) The danger model: a renewed sense of self. Science 296: 301–305. 11951032 10.1126/science.1071059

[43] Tomalka J, Ganesan S, Azodi E, Patel K, Majmudar P, et al (2011) A novel role for the NLRC4 inflammasome in mucosal defenses against the fungal pathogen *Candida albicans* . PLoS Pathog 7: e1002379. 22174673 10.1371/journal.ppat.1002379 PMC3234225

[44] De Bernardis F, Amacker M, Arancia S, Sandini S, Gremion C, et al (2012) A virosomal vaccine against candidal vaginitis: immunogenicity, efficacy and safety profile in animal models. Vaccine 30: 4490–4498. 22561143 10.1016/j.vaccine.2012.04.069

[45] Ibrahim AS, Luo G, Gebremariam T, Lee H, Schmidt CS, et al (2013) NDV-3 protects mice from vulvovaginal candidiasis through T- and B-cell immune response. Vaccine 31: 5549–5556. 24063977 10.1016/j.vaccine.2013.09.016 PMC3866209

